# Assessing the Role of STAT3 in DC Differentiation and Autologous DC Immunotherapy in Mouse Models of GBM

**DOI:** 10.1371/journal.pone.0096318

**Published:** 2014-05-07

**Authors:** Hikmat Assi, Jaclyn Espinosa, Sarah Suprise, Michael Sofroniew, Robert Doherty, Daniel Zamler, Pedro R. Lowenstein, Maria G. Castro

**Affiliations:** 1 Departments of Neurosurgery and Cell and Developmental Biology, University of Michigan Medical School, Ann Arbor, Michigan, United States of America; 2 Department of Molecular and Medical Pharmacology, David Geffen School of Medicine, UCLA, Los Angeles, California, United States of America; 3 Department of Neurobiology, David Geffen School of Medicine, UCLA, Los Angeles, California, United States of America; Cedars-Sinai Medical Center, United States of America

## Abstract

Cellular microenvironments, particularly those found in tumors, elicit a tolerogenic DC phenotype which can attenuate immune responses. Central to this process is the STAT3-mediated signaling cascade. As a transcription factor and oncogene, STAT3 promotes the expression of genes which allow tumor cells to proliferate, migrate and evade apoptosis. More importantly, activation of STAT3 in tumor infiltrating immune cells has been shown to be responsible, in part, for their immune-suppressed phenotype. The ability of STAT3 to orchestrate a diverse set of immunosuppressive instructions has made it an attractive target for cancer vaccines. Using a conditional hematopoietic knockout mouse model of STAT3, we evaluated the impact of STAT3 gene ablation on the differentiation of dendritic cells from bone marrow precursors. We also assessed the impact of STAT3 deletion on phagocytosis, maturation, cytokine secretion and antigen presentation by GM-CSF derived DCs *in vitro*. In addition to *in vitro* studies, we compared the therapeutic efficacy of DC vaccination using STAT3 deficient DCs to wild type counterparts in an intracranial mouse model of GBM. Our results indicated the following pleiotropic functions of STAT3: hematopoietic cells which lacked STAT3 were unresponsive to Flt3L and failed to differentiate as DCs. In contrast, STAT3 was not required for GM-CSF induced DC differentiation as both wild type and STAT3 null bone marrow cells gave rise to similar number of DCs. STAT3 also appeared to regulate the response of GM-CSF derived DCs to CpG. STAT3 null DCs expressed high levels of MHC-II, secreted more IL-12p70, IL-10, and TNFα were better antigen presenters *in vitro*. Although STAT3 deficient DCs displayed an enhanced activated phenotype in culture, they elicited comparable therapeutic efficacy *in vivo* compared to their wild type counterparts when utilized in vaccination paradigms in mice bearing intracranial glioma tumors.

## Introduction

Constitutive activation of signal transducer and activator of transcription-3 (STAT3) has been implicated as a central mechanism of tumor-induced immunosuppression. Activators of STAT3 include tumor-secreted factors such as IL-10, IL6, EGF, FGF, and VEGF in addition to intracellular molecules such as Src kinase and breast tumor kinase [1]–[4]. Not surprisingly, aberrant expression of STAT3 has been documented in the majority of advanced malignancies and cancer cells in culture [5]–[8]. As a transcription factor, STAT3 mediates the expression of genes such as Cyclin-D, Bcl-xl, and survivin, which promote the growth and survival of individual tumor cells [9], [10]. In addition to regulating proliferation and apoptosis, transcriptional products of STAT3 facilitate the establishment of an immune-suppressed microenvironment, thereby promoting tumor progression [3]. Wang et al. demonstrated the increased secretion of pro-inflammatory cytokines and chemokines such as TNF-α, IL-6, RANTES, and IFN-β in B16 melanoma cells after transfecting a dominant negative mutant of STAT3 [11]. These factors have pleiotropic immune stimulatory activity and are critical for inducing the activation and migration of dendritic cells (DCs). On a similar note, hyperactivation of STAT3 in CT26 or C6 tumor cells was implicated for the abnormal differentiation of DCs in cultures containing conditioned media [12].

Embryonic lethality associated with targeted deletion of the STAT3 gene in mice has prompted the development of conditional STAT3 knockouts [13]–[15]. Transgenic mice deficient for STAT3 in their hematopoietic system can develop a lethal form of colitis as result of chronic gut inflammation, demonstrating the importance of STAT3 in sequestering immune cell activation [15]. These conditional knockout models have been utilized to better understand the regulatory function of STAT3 in DCs. Using the Mx1-Cre system to ablate STAT3, Kortylewski et al. demonstrated a suppressive activity of STAT3 signaling in dendritic cells [13]. While the number of splenic DCs in STAT3 null mice was unaffected, production of IL-12 was increased in response to LPS compared to wild type [15] DCs. Furthermore, OT-II CD4^+^ T cells proliferated more in response to antigen presented by STAT3 deficient DCs [13]. NK cells isolated from STAT3 null mice bearing B16 tumors also exhibited enhanced cytotoxicity compared to WT counterparts. Not surprisingly, the growth of B16 and MB49 flank tumors was restricted in STAT3^−/−^ mice. These observations support the notion that STAT3 signaling contributes to the impaired activation of DCs and other immune cell lineages imparting a survival advantage to tumor cells.

The use of autologous DCs as cancer immunotherapies has been evaluated in a number of clinical trials and has received approval by the FDA as a treatment modality for prostate cancer [16]. The use of autologous *ex vivo* pulsed DCs in patients diagnosed with GBM has also been deemed feasible and well tolerated with encouraging clinical responses [17], [18]. Administration of primed DCs to GBM patients was associated with the induction of measurable systemic CTL responses and modest increases in survival. The limited therapeutic success of DC immunotherapy has been attributed to suppression of the immune system as consequence of malignant progression.

In light of these findings, inhibition of STAT3 has been evaluated in only a handful of immunotherapies with promising initial results [19], [20]. Using an inducible hematopoietic STAT3 knockout mouse model, we assessed the role of STAT3 in the differentiation and function of DCs generated *in vitro* by Fms-like tyrosine kinase-3 ligand (Flt3L) or granulocyte macrophage colony-stimulating factor (GM-CSF). Our results demonstrate a requirement of STAT3 for Flt3L mediated DC differentiation but not GM-CSF. Although STAT3 was not required for the expansion of DCs by GM-CSF, it did regulate their maturation. DCs deficient for STAT3 matured faster and exhibited enhanced secretion of cytokines after TLR9 stimulation. In addition allogeneic and antigen specific proliferation of T cells was increased when STAT3 null DCs were used as antigen presenting cells. We also aimed to determine if STAT3 deletion would elicit an improved therapeutic response in mice bearing intracranial GL26 tumors vaccinated with primed DCs. Although our results demonstrate a function of STAT3 in modulating DC activity *in vitro*, we found no differences in the induction of anti-tumor immune responses elicited by WT or STAT3 null DCs. Taken together, these results suggest that therapeutic efficacy of dendritic cell-mediated vaccination approaches rely not only on effective antigen presentation, but also on highly effective anti-tumor T cell-mediated responses, which may not be influenced by STAT3 signaling.

## Results

### Inducible hematopoietic STAT3 knockout mouse model

**Figure 1:**
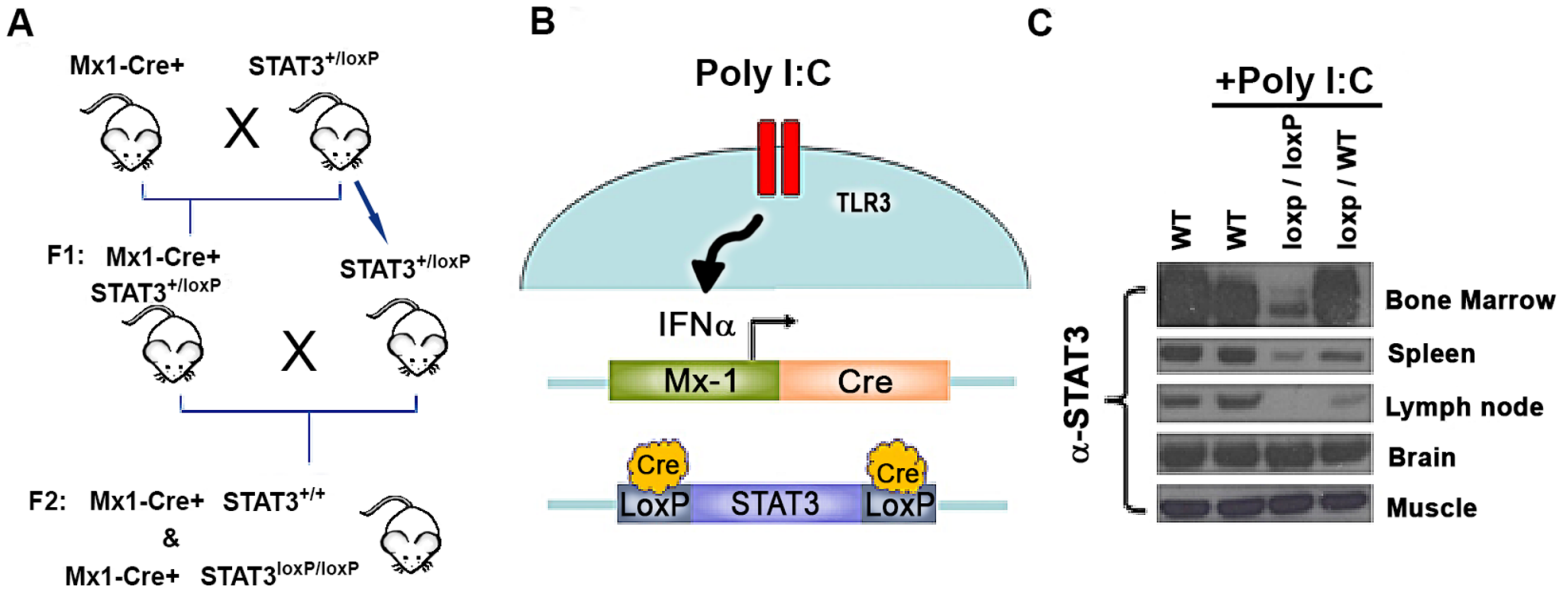
Mx1-Cre mediated excision of the STAT3 gene to generate a conditional KO mouse model. **A.** The mating scheme by which the STAT3 knockout mice were generated is depicted. **B**. Diagram illustrating the induction of Cre expression and subsequent recombination after Poly I:C administration. **C.** Western blot to assess STAT3 protein expression levels within various tissues, 2 weeks after poly I:C administration from WT, floxed and heterozygous mice.

To circumvent issues of embryonic lethality associated with total ablation of the *STAT3* gene, an inducible conditional knockout was generated in mice using the *Mx1-Cre* system as illustrated in figure 1A and previously described [13], [21]. Briefly, transgenic mice harboring a floxed *STAT3* allele [22] were crossed with mice that express Cre recombinase under the control of the IFN-inducible Mx1 promoter. The heterozygous F1 progeny was then backcrossed to parental STAT3 floxed mice to generate homozygous pups that are *Mx1-Cre/STAT3^+/+^* and *Mx1-Cre/STAT3^loxP/loxP^* (Figure 1A). Induction of Cre recombinase was performed by intraperitoneal administration of Poly I:C. As a synthetic RNA mimic and TLR3 agonist, Poly I:C stimulates the production of IFNα which leads to the activation of the Mx1 promoter and subsequent *Cre* transcription (Figure 1B). Mx1-Cre mediated recombination of STAT3 occurs in cells which are responsive to IFN type 1. Using the Mx1-Cre system, high levels of STAT3 knockout are observed in bone marrow cells, splenocytes, and draining lymph node cells but was not detected in brain or muscle tissue (Figure 1C) [21]. To bypass the potential inflammatory complications associated with Poly I:C administration, mice were given a 2 week rest period before being used as experimental subjects.

### The role of STAT3 signaling in DCs expansion and differentiation

**Figure 2:**
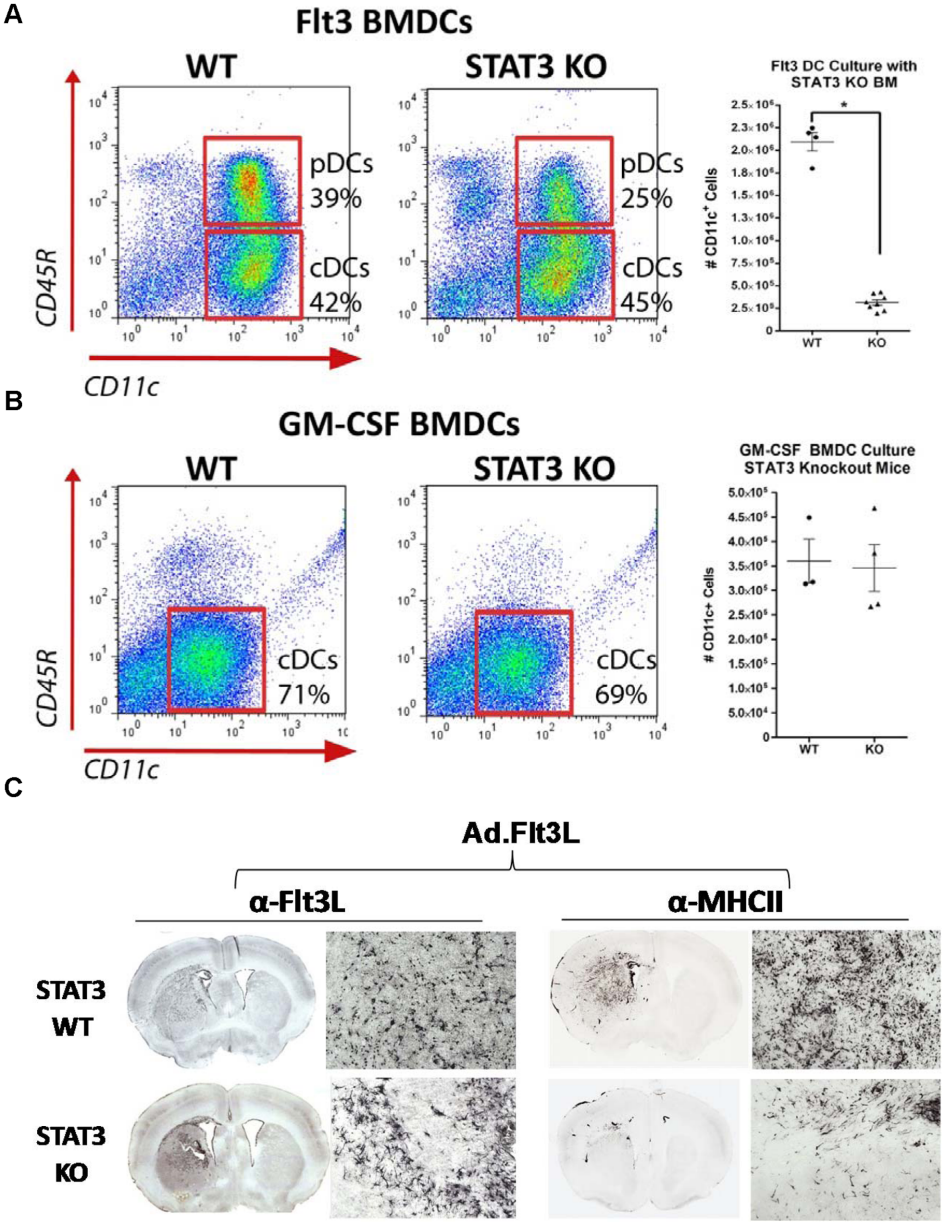
The role of STAT3 signaling in the differentiation and expansion of DCs by Flt3L and GM-CSF. **A.** WT and STAT3 null bone marrow cells were cultured in the presence of hFlt3L (100 ng/ml) for 8 days then subsequently analyzed by flow cytometry for DC subtypes. Expression of CD45R was used to distinguish pDCs (CD11c^+^/CD45R^+^) from cDCs (CD11c^+^/CD45R^−^). The total number of CD11c^+^ DCs expanded from WT and STAT3 KO bone marrow was quantified from multiple independent bone marrow cultures (*, *p*<0.05; two-tail students t-test). **B.** Flow cytometry and quantification of GM-CSF-derived (40 ng/ml) BMDCs from WT and STAT3 null bone marrow cells. **C.** WT and STAT3 deficient mice were injected intracranially with an Ad (1×10^8^pfu) that expresses human soluble Flt3-L. 8 days post injection, animals were euthanized and brains were processed for histology. Flt3 positive and MHC-II positive cells were visualized using immunohistochemistry. Mosaic micrographs of brain sections were captured at 5X and 20X magnifications.

Immature bone marrow derived dendritic cells (BMDCs) can be generated *in vitro* by culturing bone marrow cells in the presence of recombinant growth factors such as Flt3L or GM-CSF. To determine if STAT3 signaling plays a role in DC differentiation, bone marrow from WT and STAT3 knockout mice were cultured for several days in the presence of either Flt3L or GM-CSF and analyzed by flow cytometry for the presence of conventional (cDCs) and plasmacytoid DCs (pDCs). The addition of Flt3L to bone marrow cultures stimulated the expansion of loosely adherent cell clusters which were harvested for flow cytometric analysis. At day 8, 80–90% of these cells were positive for the pan DC marker CD11c. Of these cells, approximately 40% were of the pDC subtype (CD11c^+^/B220^+^) and 60% cDC (CD11c^+^/B220^−^) (*, *p*<0.05 versus WT; Figure 2A). Conversely, the vast majority of GM-CSF derived dendritic cells at day 5 were of the cDC type (Figure 2B). Interestingly, we observed a ten-fold reduction in the number of Flt3L derived DCs when the progenitor cells were deficient for STAT3. The inhibition of DC expansion did not appear to be cell type specific, as the differentiation of cDCs and pDCs was perturbed. In contrast, the absence of the STAT3 did not affect GM-CSF mediated DC differentiation as WT and STAT3 knockout cultures gave rise to an equivalent number of DCs.

Our group has demonstrated the robust infiltration of MHC-II^+^ DCs in response to intracranial injection of adenoviral vectors expressing human Flt3L [23]. To determine if STAT3 is also required for Flt3L-induced DC expansion *in vivo*, adenovirus containing the human Flt3L gene was delivered into the striatum of WT and STAT3 KO mice. Flt3L in wild type mice induced the striatal infiltration of MHC-II^+^ cells as visualized by immunohistochemistry (Figure 2C). Recruitment of MHC-II^+^ cells in STAT3 KO mice in response of Flt3L expression was highly diminished as observed with *in vitro* bone marrow cultures. Our data demonstrate a clear requirement of STAT3 for the differentiation of dendritic cells by Flt3L. In contrast, STAT3 did not appear to be required for DC differentiation via GM-CSF. In light of this data, we choose to pursue our investigation on DCs derived solely with GM-CSF.

### Phagocytosis by dendritic cells is not dependent on STAT3 signaling

**Figure 3:**
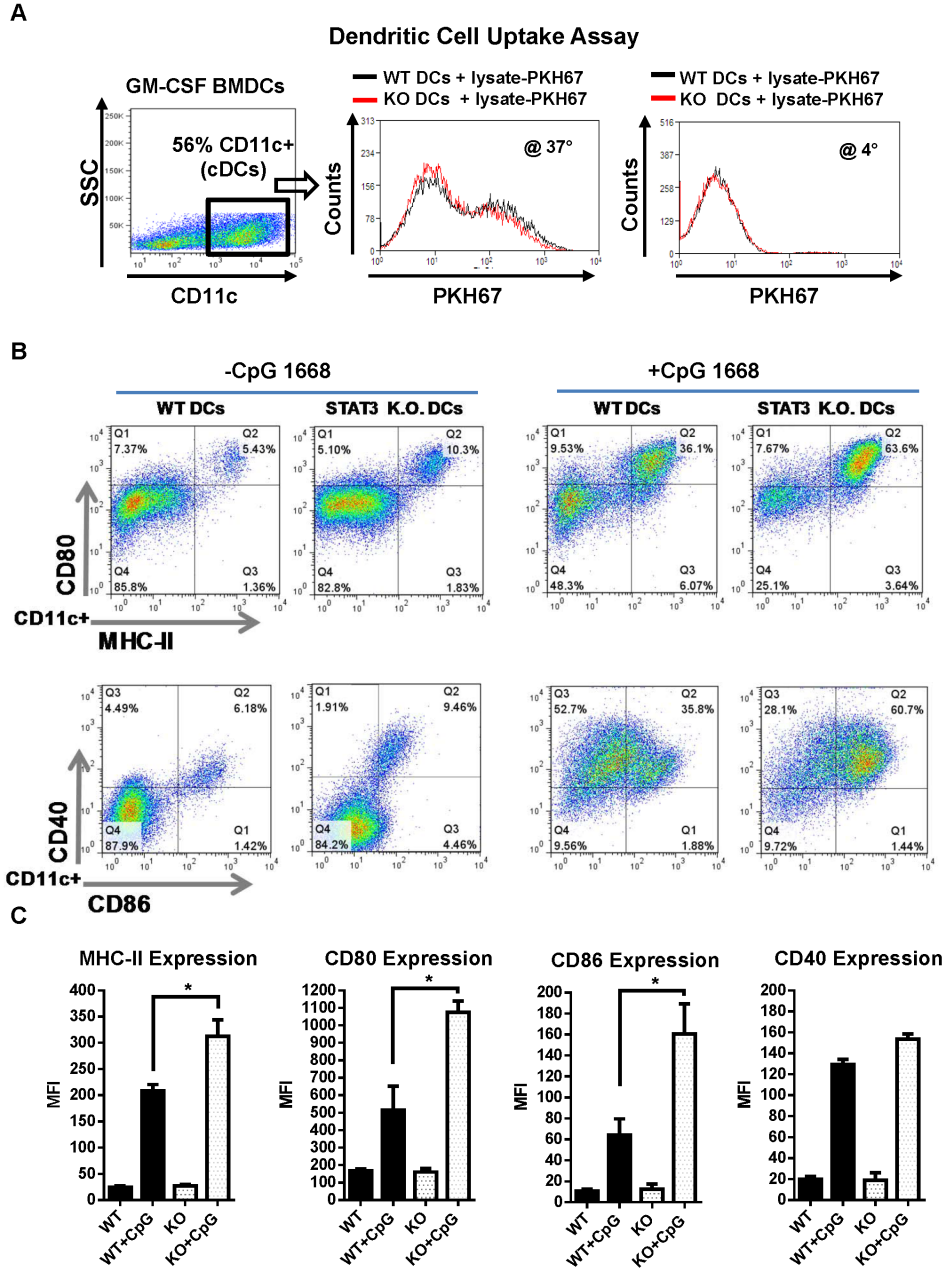
Role of STAT3 signaling on phagocytic activity and DCs' maturation. **A.** Uptake of fluorescently labeled tumor cell remnants were used as a measure of DC phagocytosis. WT and STAT3^−/−^ bone marrow cells were cultured in the presence of GM-CSF (40 ng/ml) for 6 days to expand the DC pool. BMDCs were then cultured in the presence of PKH-67 labeled GL26 tumor cell lysate for 14 hours then analyzed by flow cytometry. Fluorescence intensity of PKH-67 in CD11c^+^ cells is presented as histograms and indicative of active uptake. Phagocytosis assays were also performed at 4°C to control for uptake by means of passive diffusion. **B.** WT and STAT3 deficient BMDCs were matured *in vitro* using CpG. Cell surface expression of MHC-II, CD80, CD86, and CD40 was evaluated in CD11c^+^ GM-CSF derived DCs after an 18 hour stimulation with CpG 1668 (500 ng/ml). **C.** The median fluorescence intensity of maturation markers was quantified in two independent bone marrow cultures (*, *p*<0.05; two-tail students t-test).

The rate of DC phagocytosis is dependent on cellular subtype and maturation state. Immature DCs tend to be highly phagocytic while in more mature cells, the phagocytic capacity is repressed while antigen processing and presentation take precedence. To determine if STAT3 deletion had an impact on phagocytosis, GM-CSF derived BMDCs from WT and STAT3 knockout mice were incubated in the presence of tumor cell lysate, which was pre-labeled with the fluorescent membrane-linker dye PKH-67. After an 18 hour incubation period, flow cytometric analysis indicated that the majority of cells were positive for PKH-67 indicating robust uptake of GL26 tumor remnants (Figure 3A). Duplicate samples were also incubated at 4° to ensure that the observed uptake was an active cellular process and not some form of passive diffusion. Flow cytometric analysis indicated no statistically significant differences in the phagocytosis of tumor remnants by WT and STAT3 null DCs. Therefore, STAT3 is unlikely to play a role in the phagocytic process of bone marrow derived DCs.

### STAT3 deficient dendritic cells exhibit enhanced maturation

BMDCs derived *in vitro* using GM-CSF are typically immature, highly phagocytic, and exhibit little to no cytokine secretion. Upon recognition of danger signals or TLR agonists, DCs quickly up regulate the expression of molecules involved in antigen presentation such as MHC-II and co-stimulatory molecules CD80 and CD86, and CD40. To evaluate the role of STAT3 in the maturation process, the expression of MHC-II and co-stimulatory molecules was assessed by flow cytometry in response to the TLR9 ligand CpG 1668. The addition of CpG to GM-CSF derived DCs led to an increase in the amount of phosphorylated STAT3 at the tyrosine 705 site as indicated by Western blot, indicating a potential regulatory function in TLR signaling (Figure S1 in File S1). As expected, we observed increased expression of MHC-II and co-stimulatory molecules in response to a 12 hour treatment with CpG (Figure 3B). Interestingly, DCs deficient for STAT3 were sensitized to CpG treatment as they expressed higher levels of MHC-II, CD80, and CD86 but not CD40 compared to WT DCs. The median fluorescence intensity of MHC-II and co-stimulatory molecules was quantified from multiple animals indicating statistically significant differences in expression (*, *p*<0.05 versus WT+CpG; Figure 3C). This data suggests that STAT3 signaling can control the activation state of DCs in response to TLR agonists by inhibiting the expression of molecules involved in antigen presentation.

### Cytokine secretion by WT and STAT3^−/−^ BMDCs in response to CpG stimulation

**Figure 4:**
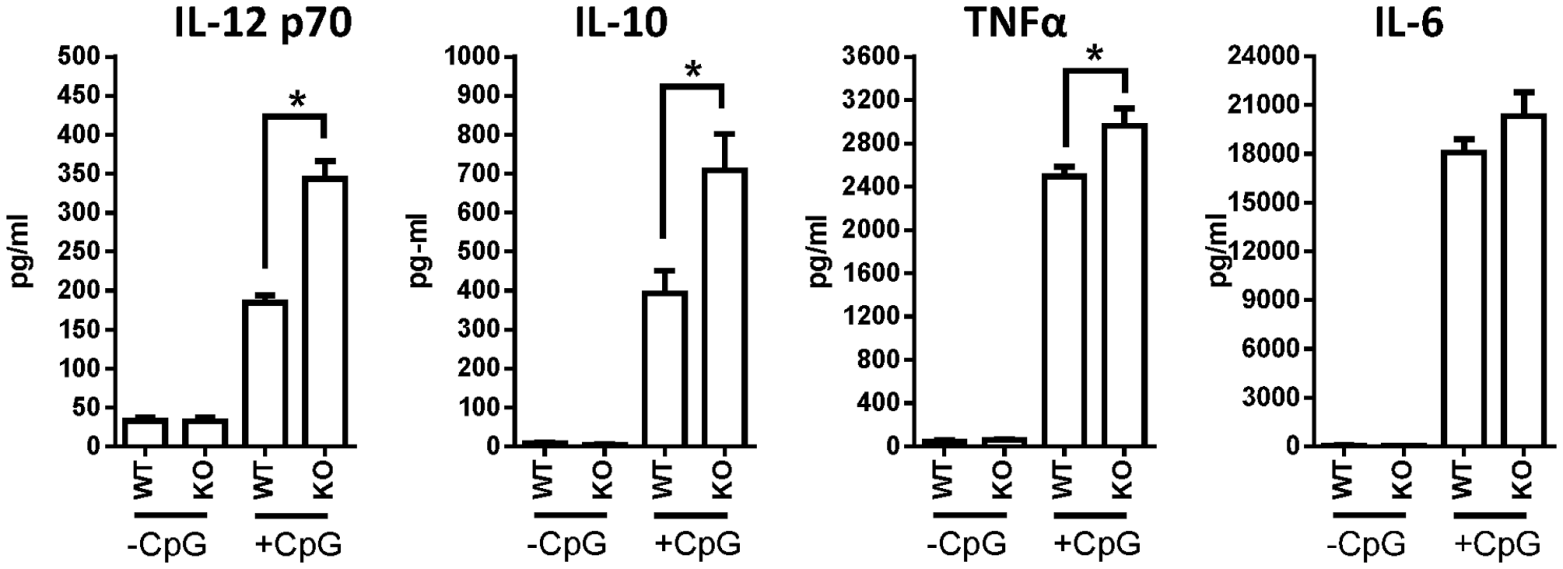
Cytokine secretion by WT and STAT3 deficient BMDCs. Secretion of IL-12p70, IL-10, TNFα, and IL-6 was measured by ELISA from supernatants of 1×10^6^ WT and STAT3^−/−^ GM-CSF BMDCs stimulated with CpG 1668 (500 ng/ml) for 18 hours in 1 ml of RPMI-10. Cytokine secretion was evaluated from 5 WT and 5 STAT3 KO mice in triplicate wells (*, *p*<0.05; two-tail students t-test).

*In vitro* CpG stimulation of WT and STAT3 null GMCSF-derived BMDCs evoked the secretion of IL-12p70, IL-10, IL-6, and TNFα into the supernatant which were quantified by ELISA. STAT3 null DCs produced roughly twice the amount of pro-inflammatory IL-12p70 compared to WT DCs after stimulation with CpG1668 for 18 hours (*, *p*<0.05 versus WT + CpG; Figure 4). Similar increases in cytokine secretion by STAT3 null DCs was observed with IL-10 and TNFα but not IL-6 (Figure 4). Deletion of STAT3 does not only increase expression of MHC-II and co-stimulatory molecules in response to TLR engagement but also leads to elevated production of inflammatory cytokines. These observations support the notion that STAT3 regulates the extent of DC activation.

### STAT3 ablation enhances antigen presentation by DCs

**Figure 5:**
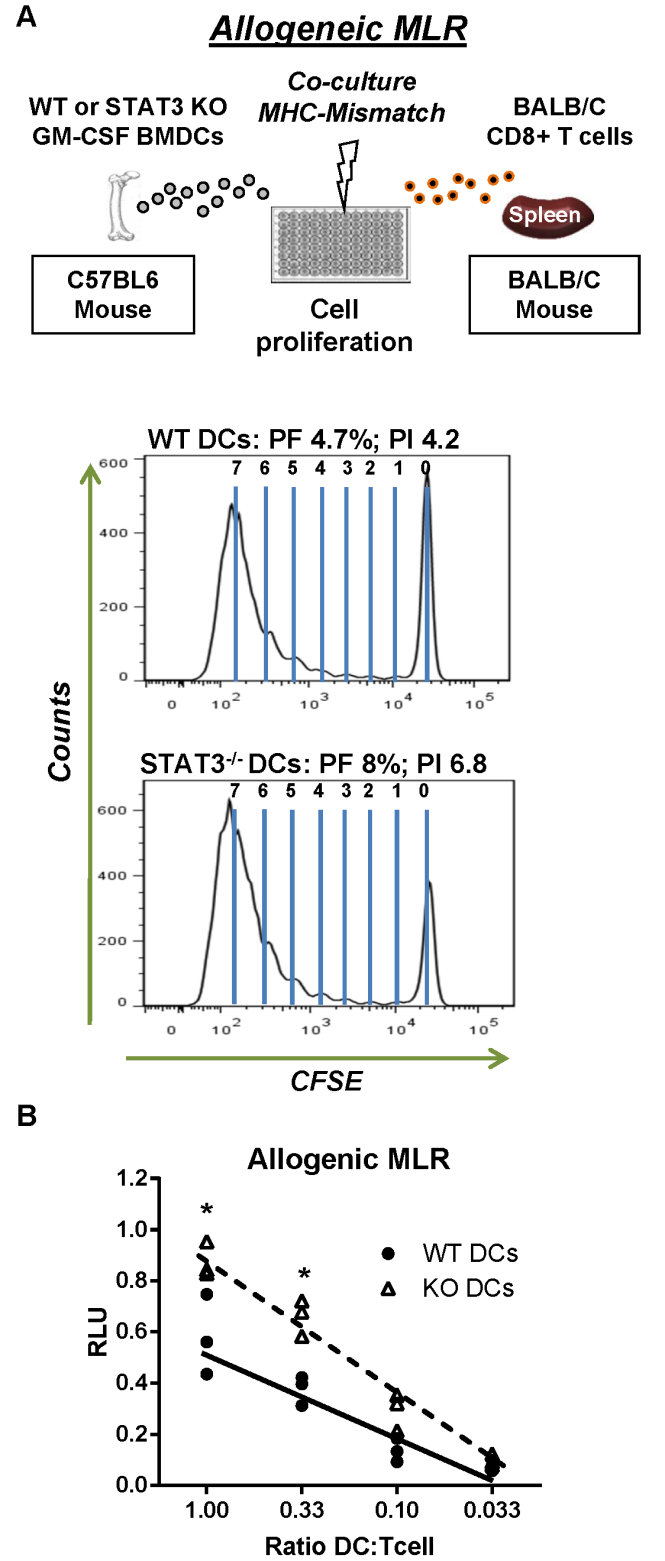
Deletion of STAT3 in DCs enhances the proliferation of allogeneic T cells. **A.** An allogeneic mixed lymphocyte reaction (MLR) was used to determine if DCs (stimulator) could induce allogeneic T cell (responder) proliferation. WT and STAT3^−/−^ DCs were derived using GM-CSF as previously outlined. BMDCs were stimulated with CpG 1668 (500 ng/ml) for 12 hours to increase cell surface expression of MHC-II prior to being γ-irradiated. To induce proliferation, 100,000 DCs were cultured with CFSE-labeled allogeneic T cells at a 1∶1 ratio in 96-well flat bottom wells for 5 days. CFSE intensity of CD8^+^ T cells at day 5 is presented as histograms. The precursor frequency and proliferation index were derived using the proliferation analysis wizard in Modfit LT computer software. **B.** CpG-matured DCs were irradiated and cultured with 100,000 allogeneic T cells at decreasing ratios of stimulator to responder in 96-well flat bottom wells. Cells were allowed to proliferate for 4 days prior to addition of the nucleotide analogue BrdU (18 hours incubation). Incorporation of BrdU into dividing DNA was determined using a colorimetric ELISA kit. 2-way ANOVA test followed by Tukey-Kramer multiple comparison test were employed to determine statistical significance (*, *p*<0.05 versus wild type).

Priming of naïve T cells by WT and STAT3 null DCs was assessed *in vitro* using allogeneic and antigen specific mixed lymphocyte reaction (MLR) assays. To assess T cell proliferation in response to allogeneic MHC mismatched DCs, CD8^+^/CD3^+^ T cells isolated from spleens of allogeneic BALB/c mice were co-cultured with irradiated WT and STAT3 null GM-CSF derived BMDCs at a 1∶1 ratio (Figure 5A). Proliferation of T cells was monitored using the carboxyfluorescein succinimidyl ester (CFSE) dye, which is routinely used to track events of cellular division. With each cellular division, the CFSE fluorescence intensity is reduced in half and is typically observed as shift of peak fluoresce on a histogram. Statistical analysis of CFSE peaks can also be employed to derive the precursor frequency (PF) and proliferative index [24] of dividing T cells. When T cells were co-cultured with allogeneic STAT3 null BMDCs, we observed nearly a two-fold increase in the number of proliferating T cells compared to cultures with WT DCs (Figure 5A). The PF of T cells dividing in response to MHC-mismatched DCs was 4.7%, and 8% for WT and STAT3 KO DCs respectively, which is in agreement of an allogeneic response. The PI was also higher when STAT3 null DCs were used (4.2 versus 6.8). Incorporation of the nucleotide analogue BrdU into proliferating allogeneic T cells was also used to confirm the results obtained with CFSE (Figure 5B).

**Figure 6:**
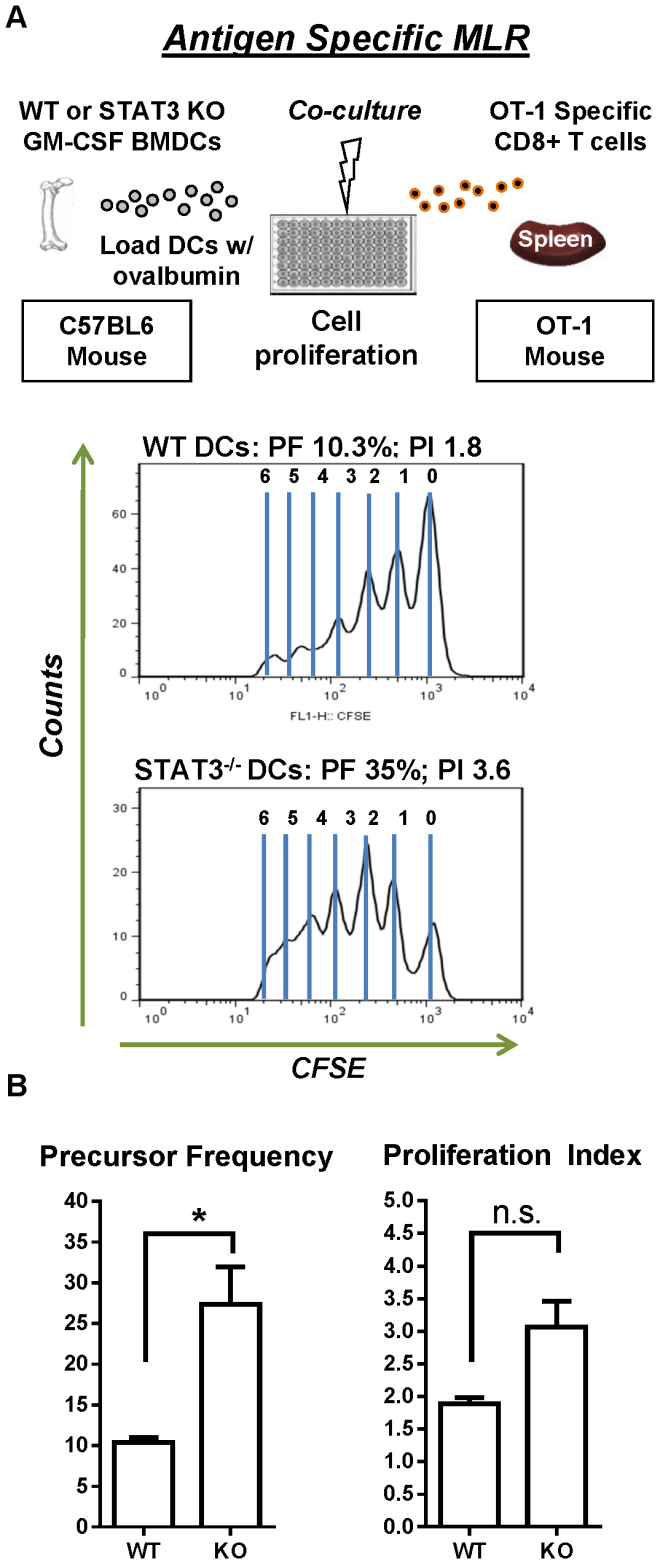
Enhanced proliferation of antigen specific T cells in response to STAT3 deficient DCs. **A.** Antigen specific MLR assay was used to assess antigen processing and presentation by GM-CSF derived BMDCs. WT and STAT deficient BMDCs were cultured with 1 µg/ml ovalbumin for 18 Hrs before being γ-irradiated. DCs were then washed of excess ovalbumin and cultured 1∶1 with 100,000 CFSE labeled OT-1 T cells for 5 days. Peaks of CFSE fluorescence were analyzed by flow cytometry on CD8^+^ OT-1 T cells. The precursor frequency and proliferation index were quantified from three separate MLR assays using non-related mice. Student's t-test was used to determine statistical significance (*, *p*<0.05 versus wild type).

Antigen specific MLR assays are good predictors of DCs' capacity to stimulate T cell responses as processing and loading of antigen peptides onto MHC are factored into the assay. To assess the impact of STAT3 deletion on antigen specific responses, DCs were cultured in the presence of ovalbumin as a source of antigen, irradiated, and cultured with antigen specific T cells isolated from splenocytes of OT-1 mice (Figure 6A). OT-1 T cells specific for the SIINFEKEL peptide found in ovalbumin underwent further rounds of division when stimulated with STAT3 null DCs compared to WT DCs (Figure 6A). Statistical analysis indicated a significant difference in the precursor frequency of diving T cells in response to WT and STAT3 null DCs (Figure 6B). Collectively, this data suggests that STAT3 null DCs are more effective at inducing T cell proliferation than their WT counterparts.

### DC vaccination elicits anti-tumor immunity in a murine GBM model which is independent of STAT3 signaling

**Figure 7:**
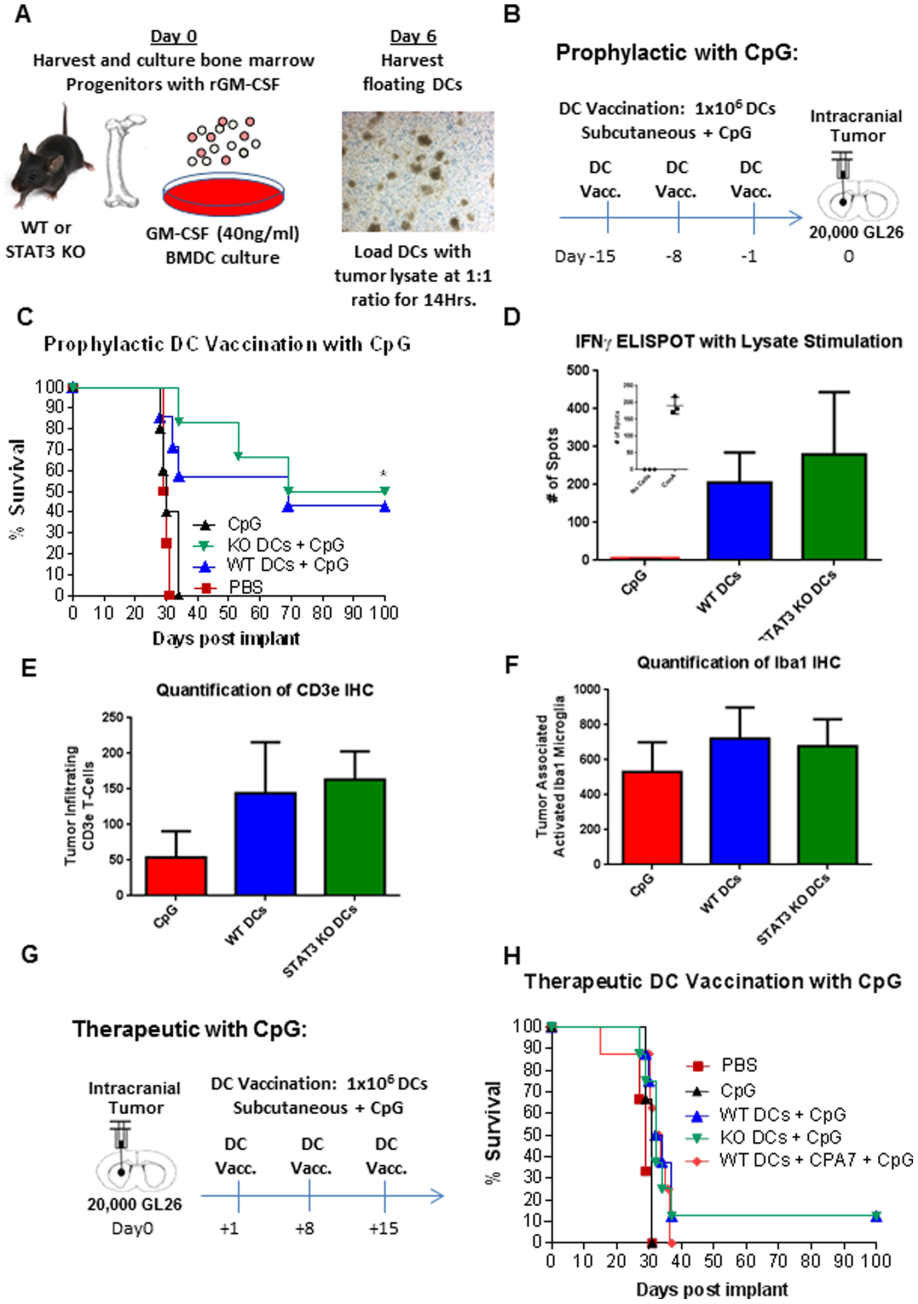
Induction of anti-tumor immunity in response to DC vaccination is independent of STAT3 signaling. **A.** Diagram illustrating the culture and priming of WT and STAT3 knockout GM-CSF derived BMDCs. Micrograph captured at day 6 of GM-CSF culture demonstrates the formation of loosely adherent cDC clusters. Tumor cell lysate was generated by subjecting GL26 cells to repeated freeze-thaw cycles in liquid nitrogen and a 37°C water bath. DCs were primed with GL26 tumor cell lysate at a 2∶1 ratio of tumor cells to DCs in RPMI-10 for 12 hours at 37°C. After loading, DCs were washed three times with PBS to remove residual tumor lysate. Loaded DCs were mixed with 30 µg of CpG 1668 immediately prior to subcutaneous vaccination. **B.** DC vaccinations were administered before (prophylactic model) tumor challenge. C57BL/6J mice were vaccinated subcutaneously with 1×10^6^ primed WT DCs (blue line, n = 5), STAT3 KO DCs (green line, n = 5), PBS-control or CpG-control on the indicated days. On day 0, mice were intracranially injected with 20,000 Gl26 glioma cells and followed for survival. **C.** Animals were monitored daily and euthanized upon signs of morbidity. Survival data is depicted as a Kaplan-Meyer curve and analyzed statistically using the Mantel log-rank test (*, *p*<0.05 versus PBS and CpG control). **D.** IFNγ ELISPOT assay was used to assess T cell IFNγ secretion from splenocytes of mice at 12 days post tumor implantation. The ELISPOT data showed a significant increase in mice treated WT DCs or STAT3 null DCs treated with CpG compared to CpG treated control mice. **E.** Quantification of CD3e immunohistochemistry showed significantly more CD3e+ T cells within both DC treatment groups compared to CpG control mice; there was no significant difference between WT DC and STAT3 null DC treated mice. **F.** Quantification of Iba1^+^ cells within the tumor showed no significant difference Iba1+ microglia between any groups. **G.** Therapeutic model of tumor bearing mouse vaccination. Mice were vaccinated subcutaneously with 1×10^6^ primed WT DCs (blue line, n = 5), STAT3 KO DCs (green line, n = 5), PBS-control on the indicated days. On day 0, mice were intracranially injected with 20,000 Gl26 glioma cells and followed for survival. **H.** Mice were monitored for survival and presented as Kaplan-Meyer survival curves. Mantel log-rank test was used to determine statistical significance (*, p<0.05 versus PBS and CpG control).

To assess the role of STAT3 signaling within autologous lysate-pulsed DC immunotherapy, mice were vaccinated using pulsed WT and STAT3 null DCs before or after the intracranial implantation of GL26 mouse glioma cells, and monitored for survival (Figure 7A and Figure S2A in File S1). The GL26 tumor model is syngeneic for C57BL/6J mice and does not express any foreign or viral antigens thereby making it a true syngeneic model. GM-CSF-derived BMDCs from WT and STAT3 KO mice were pulsed for 14 hours with GL26 tumor lysate before being washed and administered as vaccines. In the prophylactic model, mice were vaccinated subcutaneously with three doses of 1×10^6^ DCs 7 days prior to tumor challenge (Figure S2B in File S1). Intracranial gliomas were initiated 24 hours after the last vaccination by stereotactically implanting GL26 cells in the striatum. Animals vaccinated with PBS or unpulsed DCs exhibited symptoms of morbidity around day 28 and were immediately euthanized. Approximately 40% of the mice vaccinated with pulsed DCs survived long-term (>90days post tumor cell implant; Figure S2C in File S1). We did not observe any significant differences in the therapeutic response elicited by STAT3 null DCs compared to WT (Figure S2C in File S1). The induction of anti-tumor immune responses by STAT3 null DCs was also evaluated in a therapeutic treatment model in which DCs were administered post tumor cell implant (Figure S2D in File S1). This model is significantly more challenging due to pre-existing tumor burden but is also more representative of the clinical scenario. Therapeutic administration of DCs provided only a modest increase in the survival of mice bearing GL26 brain tumors and was not capable of inducing tumor regression (Figure S2E in File S1). Furthermore, as was observed in the prophylactic model, ablation of STAT3 in DCs did not enhance the therapeutic anti-tumor responses elicited by WT DCs (Figure S2E in File S1).

Our previous data indicated a role of STAT3 in regulating the response of DCs to CpG. As various experimental immunotherapies have benefited from the addition of TLR adjuvants, we tested whether co-administration of CpG would synergize with a STAT3 deficient DC-based vaccine (Figure 7B). Our results show that the addition of CpG did not significantly improve therapeutic efficacy in the prophylactic model as 40% of WT DC treated mice and 50% of STAT3 null DC treated mice survived long term (Figure 7C). IFNγ ELISPOT assay was used to assess T cell IFNγ secretion from splenocytes of treated mice 12 days post tumor implantation. The ELISPOT data showed a significant increase in IFNγ secreting T cells in mice vaccinated with WT DCs or STAT3 null DCs both treated with CpG, when compared to CpG treated control mice (Figure 7D). No significant difference between WT DC and STAT3 null DC treated mice was observed (Figure 7D). We also quantified the levels of tumor infiltrating T cells by CD3e immunohistochemistry. Our data show significantly more CD3e^+^ T cells within both DC treatment groups compared to CpG control mice; there was no significant difference between WT DC and STAT3 null DC treated mice (Figure 7E). Levels of tumor associated, activated microglia were quantified by Iba1 immunohistochemistry (Figure S3 in File S1). Our data showed no significant difference Iba1^+^ microglia between any of the groups (Figure 7F). While the addition of CpG improved the overall therapeutic response, we did not observe a discernible difference between mice vaccinated with WT DCs and STAT3 null DCs (Figure 7G and 7H).

In addition, when mice deficient for STAT3 in their hematopoietic system were challenged with GL26 cells, they became moribund at roughly the same time as WT mice bearing GL26 tumors (Figure S4 in File S1).

## Discussion

In addition to its oncogenic functions, recent studies have attributed STAT3 as a mediator of tumor-induced immunosuppression. STAT3 carries out this function by blocking the production of factors necessary for immune cell activation while simultaneously facilitating the transcription of genes that are anti-inflammatory in nature [3], [11], [12], [25]. Consequently, STAT3 has gained attention as a promising target for cancer immunotherapy. In this study, we evaluated the contribution of STAT3 to the differentiation and function of BM DCs. In addition we wanted to determine if STAT3 deletion would enhance the therapeutic efficacy of autologous lysate-pulsed DC vaccines in a mouse model of GBM. Our results demonstrate contrasting roles of STAT3 in the differentiation and function of DCs. We observed a ten-fold reduction in the numbers of Flt3L-derived DCs as a consequence of STAT3 gene ablation. These results were validated in vivo by intracranial injection of Flt3L-expressing adenovirus in STAT3 null mice. Our data supports a previously published report describing a deficiency in Flt3L-induced DC expansion as a result of Tie2-Cre mediated excision of STAT3 [26].

While STAT3 deletion had no effect on BMDCs differentiated with GM-CSF, it did appear to sensitize those cells to TLR activation. DCs deficient for STAT3 were more susceptible to CpG induced maturation, exhibiting heightened expression of MHC-II and co-stimulatory molecules in addition to elevated secretion of cytokines IL-12, IL-10 and TNFα compared to WT DCs. Furthermore, allogeneic or antigen specific T cells underwent further rounds of proliferation in response to STAT3 null DCs. These observations are in agreement with the purported anti-inflammatory functions of STAT3 and provided a rationale for exploring the use of STAT3 deficient DCs immunotherapy. To that end, WT and STAT3 null GM-CSF derived BMDCs were primed with GL26 tumor lysate and administered to mice bearing intracranial GL26 tumors. Prophylactic vaccination elicited a 40% survival rate in mice challenged with intracranial GL26 tumors. Conversely, therapeutic administration of DCs failed to elicit any long-term survivals and was only sufficient for a modest extension of life. Most important, deletion of STAT3 in DCs did not improve the efficacy of prophylactic or therapeutic DC vaccination in the GL26 mouse tumor model. A recent publication similarly showed that STAT3 knock down in DCs elicited no improvement in *in-vivo* therapeutic efficacy of monotherapy vaccination in a TC-1 murine cervical cancer model when used by itself [27]. Improved survival using STAT3 knock down DC vaccination was elicited when used in combination with bortezomib treatment via a CD8^+^ T cell response [27]. Also, the authors reported increased responses in the IFNγ ELISPOT in STAT3^−/−^ DCs, when compared to wild type DCs treated animals, the differences observed between their data and ours (Figure 7D) could be due to the fact that our model is intracranial and it is well established that immune responses elicited when tumors are located in the central nervous system could be different from tumors located in the periphery [28]–[30].

Initiating a therapeutic adaptive anti-tumor immune response is a complex process which requires the coordinated sequential activation of multiple immune cell lineages. In the context of cancer, this process becomes even more difficult as tumor cells harbor a variety of mechanisms which dampen immune cell activation. Although deletion of STAT3 in DCs is intended to bypass such signals, disruption of effector immune cells can have a big influence on the final therapeutic outcome. In addition to harboring high levels of constitutive STAT3 activation, GL26 cells produce an abundance of the immune-modulatory carbohydrate-binding protein galectin-1. A member of the lectin family, this protein regulates the growth and apoptosis of cells by mediating their interaction to the extracellular matrix and to neighboring cells [31]. Galectin-1 secretion by neuroblastoma cells was shown to induce T cell apoptosis and inhibit DC maturation [32]. Expression of galectin-1 *in vivo* by GL26 tumors could very well diminish the effector T cell pool elicited by DC vaccination. On a similar note, expression of B7-H1(PD-L1) by glioblastoma cells, stromal cells, and circulating monocytes can also induce the death of cytotoxic T cells [33], [34]. Systemic immunological defects including, but not limited to, an expansion of regulatory T cells and myeloid derived suppressor cells have been well documented in GBM patients [35], [36]. These cell types have been extensively characterized for their inhibition on T cell functions and represent yet another example of tumor-induced immune-suppression. Thus, tumors implore various mechanisms that diminish the ability of the immune system to effectively target and eliminate them.

Also, systemic inhibition of STAT3 via small molecules or conditional transgenic knockout models is not without its disadvantages. The abolishment of Flt3L-induced DC expansion as a consequence of STAT3 deletion is a potential concern, as immunotherapies designed to target STAT3 could also disrupt the biological activity of Flt3L, leading to a decrease in the number of antigen presenting cells. Using a lymphocytic choriomeningitis virus infection model, STAT3 was also shown to be required from the formation of memory T cells from CD8^+^ effector cells [37]. Based on these observations, it is clear that STAT3 functions as a pleiotropic transcription factor, regulating various aspects of cellular growth and differentiation. Although our data suggests that inhibition of STAT3 in a single cell type such as DCs is unlikely to be a fruitful strategy for boosting the efficacy of DC immunotherapy for tumors located within the central nervous system, it is possible that inhibition of STAT3 in DCs could be beneficial for *ex vivo* production of antigen specific T cells to be used for adoptive immune-therapies. Immunotherapeutic strategies which can simultaneously target multiple arms of the immune system are likely to elicit the strongest and most durable anti-tumor immune responses. Combinatorial approaches aimed at stimulating the effector T cell pool using CD25 antibody-mediated T-reg depletion or CTLA antibody stimulation in combination with DC immunotherapy have demonstrated encouraging results in mouse models of colon carcinoma and in human patients diagnosed with metastatic melanoma [38], [39]. As we gain a better understanding of the mechanisms by which tumors induce systemic immunosuppression, the development and adoption of combinatorial immunotherapies to ward off cancer will become attractive therapeutic strategies.

## Materials and Methods

### Ethics Statement

All experiments requiring the use of live animals were performed in accordance with policies and procedures outlined by the University's Committee on Use and Care of Animals (UCUCA) and Unit for Laboratory Animal Medicine (ULAM) at the University of Michigan. Animals used in experimental studies were monitored daily and euthanized at the first sings of moribund behavior. We hereby confirm that the UCUCA at the University of Michigan specifically approved all animal experiments reported in this study under protocol number PRO00001195.

### Mice, Cells, Adenovirus, Antibodies, and Reagents

GL26 glioma cells were derived from a female C57BL/6J mouse bearing a chemically induced intracranial neoplasm [39]. Female BALB/c (stock# BALB-F), C57BL/6J (stock# B6-F), and OT-1 mice (stock# 4175-F) were procured from Taconic Farms, Inc. (Hudson, NY). Adenovirus genetically engineered to express Flt3L was produced and purified in-house using procedures previously described by our lab [23]. MHC-II-eFlour 450 and its isotype were purchased from eBioscience (San Diego, CA; cat# 48-5321-82). CD80-APC and isotype were purchased from BioLegend (San Diego, CA; cat# 104714). All remaining antibodies used for flow cytometry were purchased from Becton, Dickenson and Company (Franklin Lakes, New Jersey). Mouse lymphocyte populations were detected using the following antibodies at a 1∶100 dilution: CD11c-PE (cat#553802), CD45R-PECy7 (cat#552772), CD86-FITC (cat#553691), CD8α-APC (cat#553035), and CD3ε-V450 (cat#560804). Anti-STAT3 antibody used in Western blots was procured from Cell Signaling Technology Inc. (Danvers, MA; cat# 9145). Anti-actin was purchased from Sigma-Aldrich, LLC (St. Louis, MO; cat#A1978). Poly I:C was purchased from Sigma-Aldrich, LLC [9 P1530]. Recombinant mouse GM-CSF was procured from Abd Serotec (Raleigh, NC; cat# PMP82) and recombinant mouse Flt3L was purchased from Novus Biologicals, LLC (Littleton, CO; cat# NBCI-21336). CpG 1668 ODN was synthesized by IDT (Corralville, IA).

### Inducible STAT3 knockout model

Compartmental deletion of STAT3 using the Mx1-Cre system has been described previously [15]. Transgenic mice harboring the STAT3^loxP/loxP^ alleles were generated in Dr. Sofroniew's lab (UCLA, Los Angeles CA). Mice expressing the Cre recombinase under control of the Mx1 promoter (stock #003556) were purchased from Jackson Laboratory (Ben Harbor, ME). Mx1-Cre and STAT3^loxP/loxP^ mice were mated to generate progeny that are Mx1-Cre^+^ and STAT3^fl/+^. These offspring were then backcrossed to the parental STAT3 floxed mice to yield mice which are Mx1-Cre^+^ and STAT3^loxP/loxP^. Mice were genotyped using the following set of PCR primers (STAT3For: 5′-CCTGAAGACCAAGTCATCTGTGTGAC-3′, STAT3Rev: 5′-CACACAAGC-CATCAACTCTGGTCTCC-3′, CreFor: 5′-GGACATGTTCAGGGATCGCCA GGCG-3′ and CreRev: 5′-GCATAACCAGTGAAACAGCATTGCTG-3′). Deletion of the STAT3 gene was accomplished by two IP injections, 7 days apart, of 100 µg of Poly I:C. Mice were given a two week rest period after administration of Poly I:C. Deletion of STAT3 in tissues was verified by Western blot analysis.

### Flt3 and GM-CSF bone marrow dendritic cell cultures

The femur and tibia of WT and STAT3 KO mice were removed by blunt surgical resection. The ends of the bones were cut using a razor blade to allow access to the marrow. A 10 ml syringe filled with RPMI-1640 media supplemented with 10% fetal bovine serum, glutamine, penicillin/streptomycin, and non-essential amino acids (RPMI-10) and fitted with a 26-G needle was used to flush the pulp into a 15 ml conical tube. Cells were disaggregated by repeat pipetting and centrifuged at 1,400 RPM for 5 min. The media was decanted leaving a pellet of red blood cells which were removed by resuspending in 2 mls of ice-cold ACK lysis buffer (0.15 mM NH4Cl, 10 mM KHCO3, and 0.1 mM disodium EDTA at pH 7.2) for 3 min. Cells were replenished with 8 mls of RPMI-10, centrifuged, decanted of ACK lysis buffer and resuspended in 5 mls of RPMI-10. The cell suspension was filtered through a 70 um cell strainer and counted on a hemocytometer. BMDC cultures were plated at a density of 3×10^5^ cells per well containing 40 ng/ml GM-CSF or 3×10^6^ cells per well in 100 ng/ml Flt3L. Cultures were replenished with media containing fresh cytokines on days 3 and 5 and 7. DCs appeared to be floating or lightly attached at times with sporadic clustering. GM-CSF and Flt3L BMDC cultures were harvested on day 6 and day 8 respectively for use in subsequent experiments.

### Western blots

STAT3 antibody was purchased from Cell Signaling [9 9139]. Lysates from various mouse organs were prepared by snap-freezing in liquid nitrogen followed by homogenization with a small mortar and pestle in ice-cold RIPA buffer supplemented with protease inhibitors. Protein concentration was quantified using the BCA protein assay kit (Thermo Scientific, cat# 23225). Western blotting was performed as previously described [40].

### DC Phagocytosis assay

GL26 cells were labeled with PKH-67 as recommended by the manufacturer (Sigma-Aldrich, LLC; cat# PKH67GL). Briefly, 2×10^7^ GL26 cells were washed free of serum and resuspended in 1 ml of diluent C which was provided by the manufacturer. An equal volume of diluent C containing 4 µl of PKH-67 dye was rapidly mixed with the cells (final concentration of PKH-67 was 2×10^−6^ M). After 5 min of incubation at room temperature, an equal volume of FBS was added to stop the reaction. GL26 cells were washed twice and resuspended in 500 µl using PBS. Freeze-thaw lysates of labeled GL26 cells were prepared by exposing the cell suspension to repeated cycles of liquid nitrogen and a 37°C water bath. Lysates were then clarified of large membranes and organelles by centrifugation at 2000 RPM for 10 min at 4°C. Protein concentration was quantified using the BCA assay. Phagocytosis by DCs was performed by culturing 10^6^ BMDCs in 50 µg/ml of labeled tumor lysate for 18 hours at 37°C before flow cytometric analysis of CD11c^+^ cells. Control samples were prepared with lysate at 4°C.

### ELISAs

Mouse ELISA DuoSets were purchased from R&D Systems Inc. (Minneapolis, MN), and performed according to the manufacturer's suggested protocol. Secretion of IL-12p70, IL-10, IL-6 and TNFα was measured from supernatants of 10^6^ BMDCs cultured in 1 ml of RPMI-10 and stimulated with CpG 1668 (500 ng/ml) for 18 hours ELISA measurements were performed in technical triplicates.

### Isolation of splenocytes and flow cytometry

CD8α^+^/CD3ε^+^ T cells used in the MLR assays were purified by FACS from BALB/C or OT-1 splenocytes. Splenocytes were harvested by gentle homogenization of spleens in 1 ml of RPMI-10 in 60 mm dishes. Splenocytes were then collected and centrifuged at 1,400 RPM for 3 min. The media was decanted and red blood cells were removed by resuspending the pellet in 2 mls of ice-cold ACK lysis buffer and incubating for 3 min on ice. 10 mls of RPMI-10 were then added to restore tonicity followed by another centrifugation step to yield freshly isolated splenocytes. T cells were labeled using CD3ε-V450 and CD8α-APC antibodies in cell surface staining buffer (PBS with 1% fetal bovine serum) for 20 min on ice. BMDCs were stained in a similar fashion using an alternative set of antibodies. Flow cytometry, FACS, and CFSE analysis was performed on a FACS ARIA II cell sorter (Becton, Dickenson and Company). Flow cytometry data was analyzed using Flowjo software (Tree Star Inc., Ashland, OR).

### Allogeneic and Antigen Specific MLR

Prior to culture with primed or allogeneic DCs, T cells were labeled with CFSE as follows. 3×10^6^ sorted T lymphocytes were resuspended in 500 µl of PBS. 500 µl of 10 µM CFDA, SE (eBioscience Inc.; cat#65-0850-84) was prepared fresh in PBS from a stock 10 mM solution and was mixed with cells quickly and thoroughly for a final CFSE concentration of 5 µM. Cells were placed in a dark 37°C water bath for 5 min. To halt the labeling process, 500 µl of FBS was added to the mixture and incubated for 5 min at room temperature to allow for stabilization of labeled cells. T cells were washed three times with 10 mls of RPMI-10 and resuspended at a final concentration 5×10^5^ cells/ml. WT and STAT3 deficient GM-CSF BMDCs (H2K^b^ haplotype) were generated as described in earlier sections and stimulated with CpG 1668 (500 ng/ml) for 12 hours to increase cell surface expression of MHC-II then γ-irradiated at 3000 rad to stop their growth. To induce T cell proliferation, 100,000 mature DCs were cultured with labeled allogeneic BALB/c T cells (H2k^d^- haplotype) at a 1∶1 ratio for 5 days in 96-well plates. Proliferation of allogeneic T cells in response to WT and STAT3 KO DCs was confirmed by assessing BrdU incorporation using a colorimetric ELISA kit (Roche, cat# 11647229001). Antigen specific MLR was performed by culturing immature DCs with ovalbumin (10 µg/ml) for 3 hours at 37°C. DCs were then washed twice with PBS, γ-irradiated at 3000 rad, and then resuspended at a concentration of 5×10^5^ cells/ml in RPMI-10. Splenocytes of OT-1 mice which have been genetically engineered to express TCR solely specific for the SIINFEKL peptide were used as the source of antigen specific T lymphocytes. Ovalbumin loaded DCs were co-cultured with purified OT-1 T cells for 5days at a 1∶1 ratio as in the allogeneic MLR. The PI is derived from the sum of all cells divided by the calculated number of cells in the initial population.

### Mouse glioma models and DC vaccination

Syngeneic intracranial mouse brain tumor models were generated as previously described [41], [42]. Briefly, induction of anesthesia in female C57BL/6J was performed by IP injection of ketamine (75 mg/kg) and medetomidine (0.5 mg/kg). The mice were then fitted onto a stereotactic frame for intracranial surgery. A lateral incision approximately 1.5 cm long was made exposing the skull of the animal. Using a small hand drill, a burr hole between 1–2 mm in size was made on the skull (+0.5 mm AP, +2.2 mm ML, −3.0 mm DV from bregma). 20,000 GL26 cells in 1 µl PBS were injected unilaterally into the right striatum over the course of 3 min using a 5 µl Hamilton syringe fitted with a 33-gauge needle. The needle was held in place for a further 5 min to allow cells to settle. After slowly withdrawing the needle, nylon sutures were used to close the incision site. Mice were resuscitated by IP injection of atipamezole (1 mg/kg). Analgesic buprenex was administered subcutaneously once mice began to show signs of movement (0.1 mg/kg). Prior to vaccination, 10^6^ DCs were pulsed with tumor cell's lysate equivalent to 10^6^ GL26 cells (∼60 µg). Tumor cell's lysate was prepared by resuspending 3×10^7^ GL26 cells in 1 ml of PBS and subjecting to repeated cycles of freeze/thaw as described in the DC phagocytosis assay. After an 18 hour loading period, DCs were harvested in a 15 ml conical tube, centrifuged at 1400 RPM and washed twice with 10 mls of PBS to remove excess lysate. DCs were resuspended in PBS at a density of 1×10^7^cells/ml. In the prophylactic model 100 µl of the DC suspension was injected subcutaneously into C57BL/6J mice three times 7 days apart prior to intracranial implantation of GL26 cells. When required, 30 µg of CpG 1668 was co-administered subcutaneously alongside DC vaccines. All experiments requiring the use of live animals were performed in accordance to policies and procedures outlined by the University's Committee on Use and Care of Animals (UCUCA) and Unit for Laboratory Animal Medicine (ULAM) at the University of Michigan. Animals used in experimental studies were monitored daily and euthanized at the first signs of moribund behavior.

### IFNγ ELISPOT

IFNγ immunoreactive spots were detected using the R&D Systems mouse IFN-γ ELISpot development module [9SEL485]. Briefly, 96-well PVDF plates were prepared for antibody binding by momentarily covering the wells with 35% ethanol. The wells were decanted of ethanol and washed using excess PBS. Plates were then coated overnight at 4°C with 50 µl of capture antibody. An adhesive film was used to seal the plates to prevent them from drying. The following day, capture antibody was aspirated and washed once with PBS. The plates were then incubated for 2 hours at 37°C with 100 µl of RPMI containing 10% FBS (RPMI-10) as a blocking step. Splenocytes were prepared from spleens as described in the MLR assays from vaccinated mice 12 days after tumor cell implantation. Triplicate wells of 500,000 splenocytes were cultured in 200 µl of RPMI-10 with tumor cells' lysate stimulation (GL26 tumor cell lysate; 10 µg/ml). Cells were incubated for 48 hours in a 37°C humidified incubator to allow for cytokine secretion. Cell suspensions were decanted and the plates were washed 5 times using wash buffer (0.05% Tween-20 in PBS). After splenocyte stimulation, 50 µl of biotinylated detection antibody in dilution buffer (0.2 micron filtered PBS with 1% BSA) was added for an overnight incubation at 4°C. Plates were then washed once more using wash buffer to remove excess antibody. Streptavidin-AP was used to for detection and development according to the manufactures' suggested recommendations (R&D systems, Minneapolis, MN). Following 2-hour incubation with Streptavidin-AP, 100 ul of BCIP/NBT chromogen was added to the wells and color development was allowed to take place in the dark before being washed with excess ddH20. IFN-γ spots were counted using an automated ELISPOT plate reader.

### Immunohistochemistry

Animals were anesthetized by IP administration of Ketamine (75 mg/kg) Dexmedetomidine (0.5 mg/kg). Once non-responsive the peritoneum and thoracic cavity were cut open to expose the heart. Transcardial perfusion with Tyrode's solution for 10 min to flush blood, followed by 4% PFA for 5 min to fix tissue via aluminum hub blunt needle (20GA×1 ½″) insertion into left ventricle and incision into right atrium. Brains were harvested and kept in 4% PFA at 4°C for two days. Brains were then transferred to PBS and sectioned using Leica VT1000S vibratome at 50 µm.

Free-floating sections were incubated in TBS-TritonX100 0.05% (TBS-Tx) for 30 min to permeabilize tissue. Antigen retrieval was carried out by incubating in 90°C 0.1 M sodium citrate for 20 min, followed by three 5 minute TBS-Tx washes. Sections were blocked in 10% horse serum/TBS-Tx overnight at 4°C then incubated with Rabbit anti-Mouse Iba1 primary antibody at 1∶1,000 dilution in 1% horse serum/TBS-Tx for 48 h at 4°C. Sections were then washed for 5 min, six times in TBS-Tx. Sections were incubated with biotinylated goat anti-hamster secondary antibody at 1∶1,000 dilution in 1% horse serum/TBS-Tx for 24 hours at 4°C. Sections were then washed for 5 min in TBS-Tx six times. Sections were incubated overnight with Vecta ABC in PBS at 4°C. Sections were then washed for 5 min six times in PBS and two times in 0.1 M sodium acetate. Chromogenic detection of peroxidase was performed using 3,3'-Diaminobenzidine as an enzyme substrate with an incubation time of 7 min. The reaction was halted with 10% sodium azide and sections are washed for 5 min twice in 0.1 M sodium acetate and twice in PBS, and were then mounted onto electrostatic slides in 5% Gelatin in PBS. Sections dehydrated in alcohol gradient, then cleared with xylene and coverslipped with DePeX Mounting Medium. Images were taken with Zeiss Axioplan 2 inverted brightfield microscope and Zeiss Axiovision software. For CD3e IHC, the same protocol was carried out, however a 1∶500 dilution was used and all TBS solutions were free of TritonX100 to avoid solubilizing trans-membrane CD3e protein.

Antibodies were used as follows. Hamster anti-CD3ε: cat# 553295, BD Pharmingen, Franklin Lakes, NJ; used at 1∶500 dilution. Rabbit anti-mouse Iba1: Cat. #019-19741, Wako, Richmond, VA; used at 1∶1,000 dilution. Biotinylated mouse anti-hamster IgG: cat# 550335, BD Pharmingen, Franklin Lakes, NJ; used at 1∶1,000 dilution. Biotinylated goat anti-rabbit IgG: cat# E0432, Dako, Brea, CA; used at 1∶1,000 dilution. Quantification of IHC was carried out by two independent counts of CD3e^+^ or Iba1^+^ cells within the tumor volume.

### Statistical analysis

Error bars represent SEM and asterisks denote statistically significant results. P-values of less than 0.05 were considered significant, unless noted otherwise. Using Graphpad Prism (Graphpad Software, Inc., La Jolla, CA), the two-tailed student's t-test was used to determine significance in ELISA measurements, IFN-γ ELISPOT measurements and IHC quantifications. CFSE peaks were statistically analyzed using ModFit LT software (Verity Software House) to determine the proliferation index and precursor frequency. Kaplan-Meier survival curves were analyzed using the Mantel log-rank test to determine statistical significance in median survival (Graphpad). 2-way ANOVA & Tukey-Kramer multiple comparison tests for MLR analysis were performed using NCSS software (NCSS LLC, Kaysville, UT).

## Supporting Information

File S1 (PDF)
