# Supplementary material for: Assessing the Role of STAT3 in DC Differentiation and Autologous DC Immunotherapy in Mouse Models of GBM
Source: PLoS One. 2014 May 7;9(5):e96318. doi: 10.1371/journal.pone.0096318 (PMC4013007; doi:10.1371/journal.pone.0096318)
Supplement: File S1 — (PDF) [file pone.0096318.s001.pdf]

Figure S1

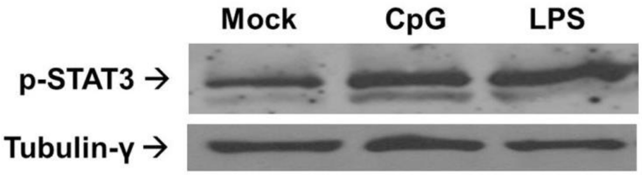

Figure S1. Phosphorylated STAT3 is increased in response to TLR stimulation. GM-CSF derived BMDCs from wild type mice were stimulated with CpG 1668 (500 ng/ml) and LPS (100 ng/ml) for 18 hours. BMDCs were then lysed using RIPA buffer to generate a whole cell lysate and probed by Western blot for expression of phosphorylated STAT3. γ-tubulin levels were used as a loading control

Figure S2

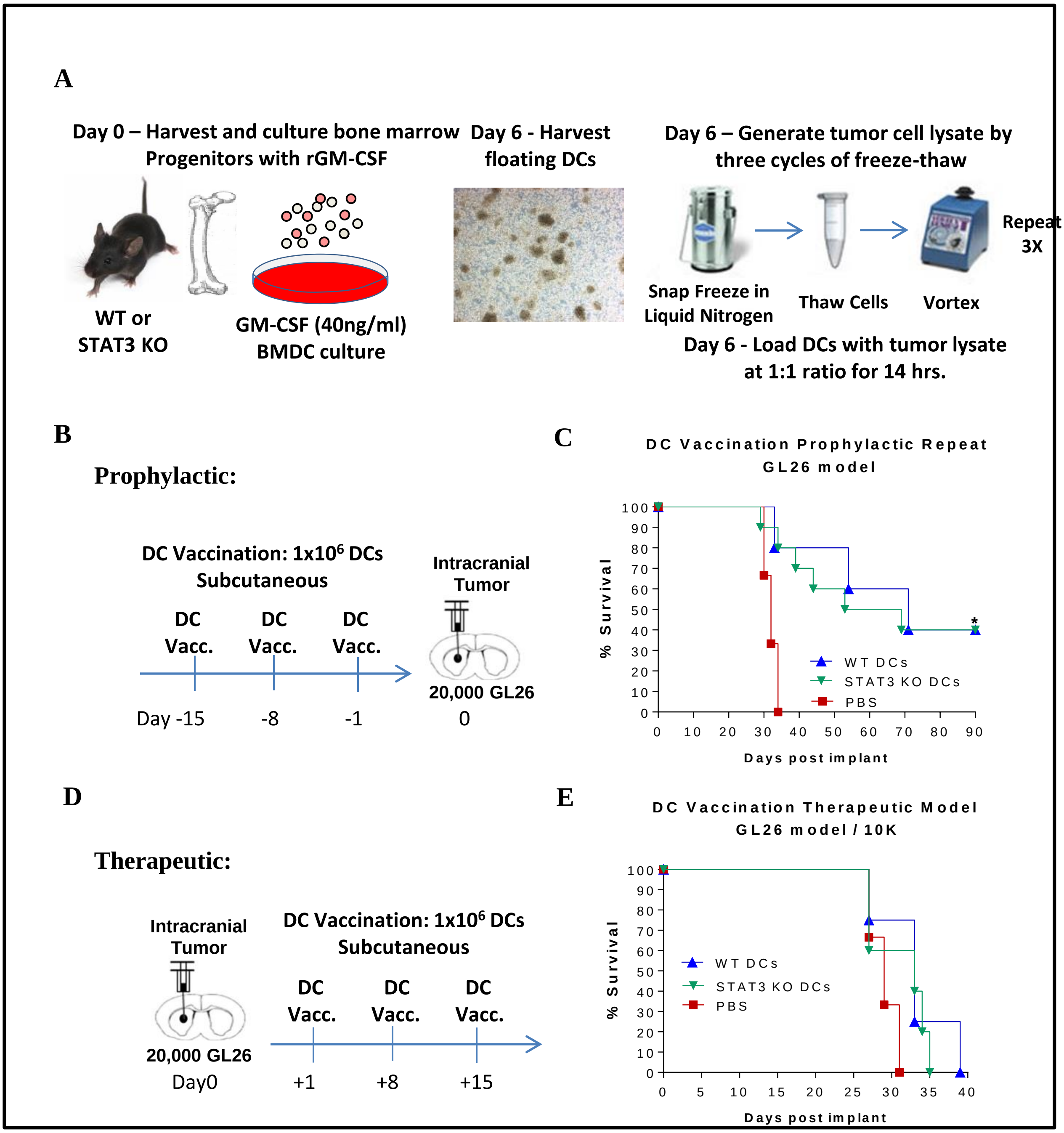

Figure S2. Efficacy of prophylactic or therapeutic autologous DC immunotherapy without CpG. A. Diagram illustrating the culture and priming of WT and STAT3 knockout GM-CSF derived BMDCs. Micrograph captured at day 6 of GM-CSF culture demonstrates the formation of loosely adherent cDC clusters. Tumor cell lysate was generated by subjecting GL26 cells to repeated freeze-thaw cycles in liquid nitrogen and a 37°C water bath. DCs were primed with GL26 tumor cell lysate at a 2:1 ratio of tumor cells to DCs in RPMI-10 for 14 hours at 37°C. After loading, DCs were washed three times with PBS to remove residual tumor lysate.B . Tumor-bearing mice were vaccinated **(B)** before (prophylactic model) or **(D)** after (therapeutic model) tumor challenge vaccinated subcutaneously with $1x10^6$ primed WT DCs (blue line, n=5), STAT3 KO DCs (green line, n=5), PBS-control on the indicated days. On day 0, mice were intracranially injected with 20,000 Gl26 glioma cells and followed for survival. **C and E.** Mice were monitored for survival and presented as Kaplan-Meyer survival curves. Mantel log-rank test was used to determine statistical significance (*, $p < 0.05$ versus PBS).

Supplementary figure 3

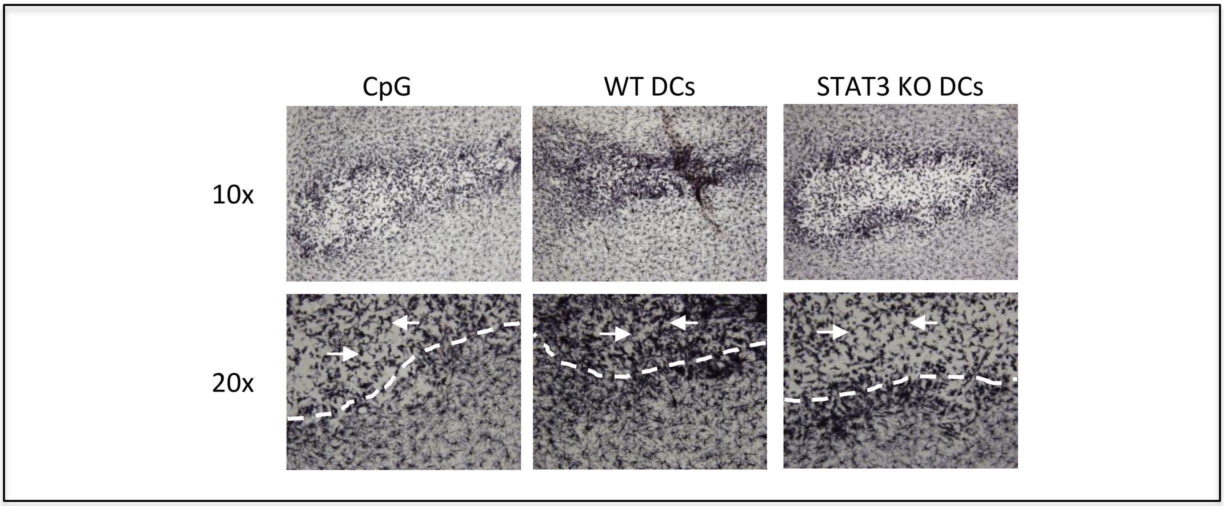

Supplementary figure 3. Iba1 Immunohistochmistry for tumor associated activated microglia quantification. Iba1 DAB peroxidase immunohistochemistry was carried out to quantify tumor associated activated microglia. A representative example of CpG control, WT DC treated and STAT3 null DC treated tumors at 12 days post implantation are shown at 10x and 20x magnification, The white dotted line indicates tumor border and the white arrows indicate examples of activated, amoeboid microglia within the tumor.

Supplementary figure 4

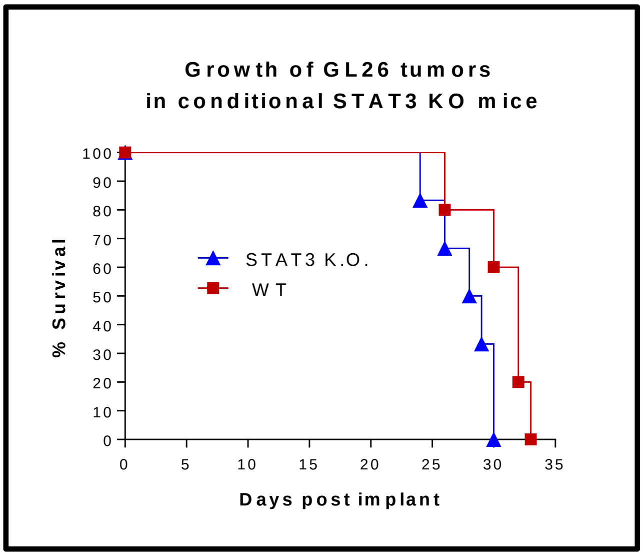

Supplementary figure 4. GL26 glioma progression in WT and conditional STAT3 KO mice. Mice deficient for STAT3 in their immune compartment, virtue of Mx1-Cre, were challenged intracranially with 20,000 GL26 glioma cells alongside wildtype controls. The mice were euthanized at first signs of morbidity and data is presented as a Kaplan-Meyer survival curve.
